# The impact of corporate violations on charitable donation behavior

**DOI:** 10.3389/fpsyg.2023.1121381

**Published:** 2023-04-25

**Authors:** Qing Zhu, Fangying Yuan, Chuming Wang, Dongqing Luan, Hong Wang

**Affiliations:** School of Management, Shanghai University of Engineering Science, Shanghai, China

**Keywords:** charitable donations, corporate violations, ownership types, analyst attention, information transparency

## Abstract

The present study investigated the impact of corporate violations on corporate charitable donation behavior and the heterogeneity influences played by corporate ownership type, analyst attention and information transparency. This study analyzed 3,715 non-financial companies in Chinese A-shares from 2011 to 2020 using panel data. The impact of corporate violations on corporate charitable donations was examined by using Ordinary Least Squares, instrumental variables two-stage least squares and propensity score matching method. Consequently, the following conclusions are presented. First, corporate violations are significantly positively correlated with the level of corporate charitable donations. Second, among the companies with high analyst attention, high information transparency, or non-state-owned nature, the positive impact of enterprise violations on charitable donations is greater. These findings suggest that charitable giving may be used by some businesses as an undesirable tool to conceal their irregularities. No study has been conducted regarding the influence of corporate violations on corporate charitable donations in China. This study is a pioneering study that seeks to give insights into the link between these variables in the context of China, which has practical implications for gaining insights into corporate charitable giving in China, as well as identifying and curbing “hypocritical” corporate charitable donation behavior.

## 1. Introduction

In recent years, natural disasters and public health emergencies have occurred frequently in China, and an increasing number of enterprises have actively participated in philanthropy and fulfilled their corporate social responsibilities through charitable donations. According to *Giving China 2020*, released by the *China Charity Alliance*, the amount of corporate charitable donations in 2020 reached 121.811 billion yuan (approximately US$ 19.28 billion), accounting for 58.39% of the total charitable donations, and it was the main source of charitable donations in China. Corporate charitable donations are becoming increasingly normalized, arousing widespread concern in academia and the public.

The discussion on the motivation behind corporate charitable donations has always been the focus of academic research. The early view was that corporate charitable donations were purely altruistic (Carroll, 1991; Ba Rrett, 2000). Recent research shows that enterprises will significantly increase their level of charitable donations in the case of financial restatement (Koehn and Ueng, 2010), increased litigation risk (Shiu and Yang, 2017) and change of government officials (Liu et al., 2021). In this case, the altruistic motivation of charitable donation lacks sufficient explanatory power. Therefore, many scholars have proposed that with the development of the capital market, a corporate charitable donation is no longer merely an altruistic behavior out of social responsibility but has gradually become a strategic tool for achieving corporate goals (Godfrey, 2005; Zhang et al., 2010; Jia and Zhang, 2011; Gautier and Pache, 2015). In some specific cases, enterprises may even use charitable donations to divert attention from or conceal their bad behavior. At this point, corporate charitable giving is primarily motivated by instrumental factors. From this perspective, charitable donations have become hypocritical (Du, 2015). Whether corporate charitable donations are made out of genuine goodwill or hypocrisy needs to be examined on a case-by-case basis from a variety of perspectives and contexts.

In fact, while companies have become increasingly active in charitable giving over the last decade in China, corporate violations have become more frequent. Compared to developed Western countries, China’s capital market started late, with a lack of development and regulation, and investors are relatively immature and less able to accurately identify market information. What is the relationship between corporate violations and their charitable donations in China’s capital market? Do corporations make more giving decisions after a violation? In other words, are corporate charitable giving behaviors motivated by undesirable motives to cover up their violations? Answering the above questions will have a positive effect on expanding research in the field of corporate charitable giving motives, deepening the understanding of corporate charitable giving behavior, optimizing the ecology of China’s capital market and assisting investors’ decisions.

While several recent studies have found that firms increased their giving after negative events such as declining performance (Li et al., 2016) or litigation risks (Dai et al., 2016; Fu and Ji, 2017). However, little attention has been paid to the relationship between corporate violations and the level of corporate charitable giving, and there is no study on the impact of corporate violations on corporate charitable giving in China.

Therefore, in order to reveal the hidden instrumental motives behind corporate charitable giving in China’s capital markets and provide empirical evidence to strengthen market regulation and investor decision-making, this paper examines the impact of corporate violations on the level of corporate charitable donations using a sample of 3,715 Chinese A-share non-financial listed companies from 2011 to 2020. The study finds that the level of charitable donations will significantly increase after companies violate the rules, indicating that some companies may use charitable donations as a tool to conceal their violations. Additionally, we further examine the heterogeneous impact of corporate irregularities on the level of corporate charitable donations under different ownership types, analyst attention and information transparency.

The possible research contributions of this paper are as follows. First, the existing literature mainly studies corporate violations from the perspectives of influencing factors and economic consequences (Kedia and Rajgopal, 2011; Chava et al., 2018; Hogarth et al., 2018), with less attention to the charitable donations of violating companies. Although several studies have found that corporations increased their charitable giving after certain negative events (Gu et al., 2016; Liu and Chen, 2018), there is no research on the impact of corporate violations on corporate charitable giving in China’s capital market. We systematically examine the impact of corporate violations on the level of charitable donations, revealing the hidden motives of corporate participation in charitable donations from the perspective of concealing violations, thus extending the research on violations in the field of social responsibility and expanding the existing research perspective. Second, this work is based on panel data from a developing country, namely China, unlike the previous studies that examined developed countries (Koehn and Ueng, 2010). This study’s outcome is expected to deepen the understanding of corporate charitable donations in China’s capital market and shed new light on optimizing the ecology of China’s capital market and assisting investors’ decisions. Furthermore, we incorporate the ownership type, analyst attention and information transparency into the research scope which is expected to enrich relevant research in the field of corporate charitable donation motives and provide a theoretical basis for subsequent studies while facilitating the identification and containment of “hypocritical” corporate giving behavior.

The remainder of this paper is organized into several sections. Section 2 and Section 3, respectively, presents the literature review and the theoretical analysis. This is followed by the outline of the research methodology in Section 4, the regression results and discussion in Section 5, as well as the heterogeneity analysis in Section 6. Lastly, Section 7 presents the overall conclusion of this study.

## 2. Literature review

In general, corporate violations include operational misbehavior, information disclosure violations, as well as capital market misconduct such as share price manipulation. Scholars have conducted research on corporate violations mainly from the perspectives of influencing factors and economic consequences. In terms of influencing factors, many studies have shown that a good internal and external corporate governance environment can inhibit corporate violations; otherwise, it may provide opportunities. Uzun et al. (2004) found that with an increase in the number of independent directors on the board of directors, as well as the audit and compensation committees, the probability of a company’s violations decreased significantly. Kedia and Rajgopal (2011) believed that strong government supervision will increase the cost of non-compliance and reduce the tendency of enterprises to violate regulations. Miller (2006) found that the media can expose a company’s financial fraud and irregularities by obtaining and disseminating information, thereby reducing the occurrence of corporate irregularities.

In terms of the economic consequences of enterprise violations, the existing literature shows that the investigation and punishment of corporate violations can seriously affect the level of trust and support of suppliers, investors, consumers and other stakeholders in the corporation, making them doubt the company’s operating conditions and integrity, which in turn undermines the cooperative relationship between the company and its stakeholders and has a seriously negative impact on the company in many ways, specifically by reducing the company’s reputation, increasing its financing costs (Klein and Leffler, 1981; Chen et al., 2011), resulting in lower share prices and reduced profits (Karpoff and Lott, 1993; Johnson et al., 2014).

The donation behavior is one aspect of corporate social responsibility. The pyramid model of Carroll (1991) divided CSR into four components: economic responsibility, legal responsibility, ethical responsibility and charitable responsibility. The first three are mandatory responsibilities, while charitable responsibility is at the top of the pyramid because of its optional nature. Recently, a growing number of scholars have taken an interest in the field of corporate charitable giving.

Studies have shown that charitable donation has a positive effect on improving business-stakeholder relations and promoting business growth. According to stakeholder theory, stakeholders such as customers, suppliers, government and financial institutions hold the resources that companies need to develop (Donaldson and Preston, 1995) and play an important role in the operation and growth of the company. On the one hand, due to its non-compulsory nature, charitable donations can greatly reflect the benevolence and social responsibility consciousness of enterprises, forming and enhancing their moral capital and generating a stronger social effect than other corporate social responsibility activities. Godfrey (2005) found that corporate social responsibility activities generated positive moral capital, which acts as a relational intangible asset for the company, effectively acting as an insurance policy and increasing corporate value. On the other hand, since donations represent an outflow of cash that cannot be implemented unless there are relatively abundant disposable cash resources (Li et al., 2016), charitable donations can be a signal that the company is in a good financial position (Shapira, 2012; Lys et al., 2015). This in turn can increase stakeholder confidence in the company’s future performance.

Regarding the motivation behind charitable donations, relevant studies have obtained relatively rich research results. Early studies stated that charitable donations by enterprises were mainly to fulfill social responsibilities and meet the expectations of stakeholders, which was a simple altruistic behavior. With the development of the capital market, the competition among enterprises has become increasingly fierce. An increasing number of studies show that enterprises tend to regard charitable donations as a strategy for achieving their own development goals (Campbell, 2007) —establishing a good brand image and enhancing the social reputation of enterprises through charitable donations (Brammer and Millington, 2005; Gardberg and Fombrun, 2006; Wang and Qian, 2011). By virtue of charitable donations, corporations can send a signal of good business conditions and abundant cash flow to the outside world (Lys et al., 2015; Chang et al., 2018) to enhance enterprise value (Su and He, 2010; Hogarth et al., 2018), establish or maintain government enterprise relations through charitable donations (Hao et al., 2020) and obtain more government subsidies (Parish, 2006; Jia et al., 2019; Liu et al., 2021).

Scholars have also noted the hidden instrumental motives behind corporate charitable donations, arguing that in certain adverse situations, companies may use charitable giving as a tool to cover up negative information, divert external attention, improve corporate image and defuse corporate crises (Godfrey, 2005; Jia and Zhang, 2011). For example, enterprises with financial restatement events often improve their corporate image by improving their level of charitable donations (Koehn and Ueng, 2010), while some enterprises also respond to litigation risks by increasing the number of charitable donations (Shiu and Yang, 2017). Some studies provide evidence that making donations can help cover up negative events or divert public attention from corporate misconduct (Campbell, 2007; Koehn and Ueng, 2010). In addition, several recent studies have confirmed the role of charitable giving in repairing corporate reputation, in other words, preventing further damage to their reputation by making charitable donations after certain negative events (Li et al., 2016; Xia et al., 2019). Studies also show that when faced with a negative event, higher donations are associated with lower share price reductions (Godfrey et al., 2009; Minor and Morgan, 2011; Shiu and Yang, 2017). Corporate violation, which includes operational misbehavior, information disclosure violations, as well as misconduct in the capital market, can be much more damaging to a company’s reputation than the negative events described above, such as poor environmental performance and employee welfare. Moreover, as China’s capital market continues to progress and improve, the government is gradually improving its regulatory system, with increasing regulation and the probability of corporate violations being detected increasing significantly, so there may be a strong incentive for corporations to enhance their reputation and play an insurance role through charitable donations following violations in the Chinese capital market.

The above-mentioned literature shows that domestic and foreign scholars have conducted abundant research on corporate irregularities and corporate charitable donation motives but have rarely paid attention to the hidden motives of corporate charitable donations in the context of violations. The existing literature shows that under certain circumstances, corporate charitable donations can effectively play an instrumental role in diverting outside attention and concealing bad information. From this perspective, it is essential to comprehensively investigate the possible changes that may ensue regarding the level of charitable donations of enterprises after violations occur. Therefore, this study combines corporate violations with corporate charitable donations and examines the impact of corporate violations on the level of corporate charitable donations. Further, it investigates the role of ownership type, analyst attention and information transparency to deepen the research on the instrumental motivation behind corporate charitable donation behavior.

## 3. Theoretical analysis and research hypothesis

Stakeholder theory holds that the essence of an enterprise is the contract aggregate of stakeholders, and the survival and development of any enterprise are inseparable from the input and participation of several stakeholders (Freeman, 1984). As a typical negative incident, once a company’s violations are investigated and punished, the company’s reputation and the contractual relationship between the company and its stakeholders will be significantly damaged, thereby sending negative signals, such as poor management and poor development prospects, to the outside. Furthermore, it will seriously damage investors’ confidence, ultimately leading to higher financing costs and lower stock prices (Firth et al., 2011; Chava et al., 2018). Therefore, enterprises have the motivation to divert the attention of the outside world to enterprise violations through other enterprise behaviors to minimize and compensate for the losses caused by enterprise violations.

Corporate charitable donations can play a positive role in many aspects, thus providing a tool for companies to deal with violation crises. On the one hand, according to stakeholder theory, actively fulfilling social responsibilities is one of the ways for enterprises to meet the ethical expectations of stakeholders and strengthen the contractual relationship between enterprises and stakeholders (Freeman, 1994). Because it is non-mandatory, direct and relatively difficult to imitate (Du, 2015), charitable responsibility is the highest level of the corporate social responsibility pyramid (Carroll, 1991). Therefore, compared with other social responsibility behaviors, charitable donations are more likely to be reported by the news media and the public’s attention. This will send a positive signal to the outside world that the company is financially sound and has bright development prospects (Shapira, 2012), thereby enhancing the confidence of investors, suppliers, customers, employees and other stakeholders in the sustainability of the business and helps the company to reap the benefits of a good capital market response (Lys et al., 2015; Chang et al., 2018; Young-Chul and Tai-Young, 2019). Research has shown that corporate social responsibility activities influence consumers’ purchasing decisions (Crampton and Patten, 2008). With the continuous development of the market economy as well as the change and upgrading of consumer attitudes, consumers tend to choose conscientious companies with a high level of ethics and social responsibility when selecting products or services (Bhardwaj et al., 2018). Actively practicing philanthropy, as a visual manifestation of consciously fulfilling social responsibility, will effectively enhance the social status and reputation of enterprises, and establish and maintain consumer preference and favor for their products and services (Godfrey, 2005). On the other hand, “instrumental theory” indicates that enterprises can transfer the public’s attention to their negative events to their excellent social responsibility performance by fulfilling social responsibilities, thereby reducing the damage of negative information to the corporate image (Peloza, 2006). As the most intuitive and representative act of social responsibility, the reputational insurance effectiveness and instrumental motivation of corporate charitable donation have also been validated in many studies. For example, under certain unfavorable circumstances, such as poor corporate environmental performance, high litigation risk and financial restatement, companies tend to divert public attention and reduce the impact of negative events through more charitable donations (Godfrey, 2005; Koehn and Ueng, 2010; Du, 2015). While corporate violates, which include operational misconduct, information disclosure violations and misconduct in the capital markets, can be much more damaging to a company’s reputation than the negative events described above. In summary, it is reasonable to speculate that corporations also have a strong, if not stronger, instrumental incentive to make charitable donations in the event of a breach, in order to make it a rainy day insurance and thus mitigate the severe negative impact of a corporate violation being revealed.

Additionally, from the management perspective, to maintain its reputation, salary, position and future career development, when violations occur within the enterprise, the management will take extensive measures to enhance the corporate social image of the enterprise and divert external attention to conceal relevant negative information from exposure as much as possible (Kothari et al., 2009). Extensive participation in charitable donations can transfer the public’s attention to the social responsibility of the company’s good performance (Chen et al., 2008) and prevent the outside world from discovering the company’s irregularities. Further, it can help establish a good corporate charitable image, thereby making the public imperceptibly believe that its management with a high sense of responsibility and morality will not deliberately commit immoral acts such as violations. In general, public trust in companies with higher levels of charitable giving is generally higher, meaning that the act of charitable giving can create a certain amount of intangible moral capital and provide a degree of reputational insurance for the corporate (Minor and Morgan, 2011; Xia et al., 2019). Thus, even if an enterprise is investigated and punished for violations in the future, it can minimize the pressure of external public opinion and reduce the impact of violations on management. Therefore, management has a significant incentive to use charitable donations as a self-help tool after a corporate violation—by increasing corporate charitable donations to cover up its violations as well as reduce the potential loss of benefits to management from disclosure of corporate violations. Based on the aforementioned analysis, we propose the following hypothesis:

*Hypothesis 1*: Corporate violations are significantly positively correlated with the level of charitable donations.

## 4. Research design

### 4.1. Data source and sample selection

Due to the lack of empirical evidence on the relationship between corporate violations and levels of charitable donations in China’s capital market, and given the specificity of financial sector companies, which represent a relatively small share of China, and their significant differences from companies in other sectors in terms of financial position, competitive environment, risk–reward and applicable policies, this study uses a panel data of non-financial companies in Chinese A-shares from 2011 to 2020. After removing observations with missing values and outliers for key variables, our final research sample comprised 3,715 non-financial companies, yielding 25,573 panel data. All the continuous variables were winsorized at the 1 and 99% quantiles.

We used data from the China Stock Market Accounting Research (CSMAR) database. As one of the largest databases of Chinese listed firms, the CSMAR database serves as the primary source of information on the Chinese stock market and the financial statements of listed firms (Wang and Qian, 2011). Specifically, the corporate violation data were obtained from the “CSMAR Corporate Violation and Punishment Database,” which discloses the penalties issued by the relevant authorities for corporate violations, including the time and matter of violation, the duration and the measures of the penalty, and it has the most authoritative and comprehensive data on the corporate violation that is publicly available. In recent years, China has strengthened the supervision and investigation of enterprises’ violations and has carried out retroactive investigations of many enterprises’ violations. It is worth noting that the data used in this study is for the years in which the companies committed operational misconduct, information disclosure violations and capital market misconduct, rather than the years in which they were investigated or penalized by regulators. The charitable donation data came from the “CSMAR Financial Statement Notes Database,” and they were obtained by manually screening the detailed items of “non-operating expenses” in the notes to the financial statements.

### 4.2. Variable definitions

#### 4.2.1. Dependent variable: corporate charitable donations (*Dona*)

Referring to Chen et al. (2008) and Du (2015), we measured the level of corporate charitable donations by multiplying the ratio of the amount of corporate charitable donations to the revenue by 1,000 to improve the comparability of data and eliminate scale effects and magnitude differences.

#### 4.2.2. Independent variable: corporate violation (*Fraud*)

According to Chinese regulators, corporate violations are classified into three categories: operational misconduct, disclosure violations and misconduct in the capital market. The information related to corporate violations is collated and disclosed in detail in the CSMAR database. We followed Khanna et al. (2015) and used a dummy variable of whether the corporate had violation as described above to measure the corporate violation (denoted by *Fraud*) – if the corporate committed a violation during the year according to the CSMAR database, the variable is assigned a value of 1; otherwise, it is assigned a value of 0.

#### 4.2.3. Control variable

Following the prior literature (Brown et al., 2006; Wang and Qian, 2011; Du, 2015), we controlled for factors that could potentially affect corporate violations. Specifically, we controlled for the following variables: firm size (*Size*), leverage (*Lev*), return on assets (*ROA*), cash holdings (C*ash*), board duality (*Dual*), board size (*Bsize*), board independence (*Indep*) and audit quality (*Big4*). Additionally, the industry fixed effect (*Ind*) and annual fixed effect (*Year*) were controlled for in the model. See Table 1 for the detailed variable definitions.

**Table 1:** Variable definitions.

Variable	Symbol	Definition
Corporate charitable donation	*Dona*	The amount of corporate charitable donations divided by its revenues multiplied by 1,000
Corporate violation	*Fraud*	Dummy variable which, according to the CSMAR database, takes a value of 1 if the firm committed a violation in that year and 0 otherwise
Firm size	*Size*	Natural logarithm of total assets
Leverage	*Lev*	Total liabilities divided by total assets
Return on assets	*ROA*	Net profit divided by total assets
Cash holdings	*Cash*	Cash and its equivalents divided by current liabilities
Board duality	*Dual*	Dummy variable that takes a value of 1 if the Chairman of the Board is also the Chief Executive Officer and 0 otherwise
Board size	*Bsize*	The number of board members
Board independence	*Indep*	The number of independent directors divided by the number of board members
Audit quality	*Big4*	Dummy variable that takes a value of 1 if audited by a Big Four accounting firm and 0 otherwise

### 4.3. Empirical model

To test Hypothesis 1, referring to Du et al. (2014) and Du (2015), we estimate Eq. 1 including corporate violation (*Fraud*), firm-specific control variables and industry and year dummies:


(1) $$\begin{array}{l} \text{Don}a_{i,t+1}=\beta_{0}+\beta_{1}\text{Frau}d_{i,t}+\beta_{s}\sum {\text{Control}}_{i,t}\\ \;\;\;\;\;\;\;\;\;\;\;\;\;\;\;\;\;\;\;\;\;\sum \text{Ind}+\sum \text{Year}+\varepsilon_{i,t} \end{array}$$

Considering that there may be a certain lag in the impact of corporate violations on the level of corporate charitable donations, all the explanatory and control variables in Eq. 1 were lagged by 1 year.

In Eq. 1, the dependent variable is *Dona* which is the label of corporate charitable donation. The main independent variable in Eq. 1 is *Fraud*, the label of corporate violation. A number of previous studies have documented systematic evidence that firm characteristics have important impacts on corporate philanthropy (Brown et al., 2006; Wang and Qian, 2011; Du, 2015). Therefore, we include eight firm characteristics, i.e., *SIZE*, *LEV*, *ROA*, *Cash*, *Dual*, *Bsize*, *Indep* and *Big4*, in Eq. 1. Table 1 provides variable definitions in detail. We also include industry dummies and year dummies in Eq. 1 to control the industry and year-fixed effects.

In Eq. 1, *i* represents firm, *t* represents year. If the coefficient on *Fraud* (i.e., β_1_) is positive and significant, it implies that more charitable donations are made in the year following the year in which the firm commits a breach, i.e., the empirical evidence supports Hypothesis 1.

## 5. Empirical analysis

### 5.1. Descriptive statistics and single variable inspection

Table 2 reports the descriptive statistics for the variables in Model 1. The average value of the corporate charitable donation level (*Dona*) is 0.372, indicating that for every 10,000 yuan of operating income created by the sample companies, 0.372 yuan is used for charitable donations. Further, the minimum value of *Dona* is 0, and the maximum value is 5.417, while the standard deviation is 0.843, indicating that the level of charitable donations among the sample companies is considerably different. Finally, the median value of *Dona*, at 0.058, is greater than 0, indicating that more than half of the listed companies in the sample have made charitable donations. The average value of corporate violations (*Fraud*) is 0.088, indicating that 8.8% of the listed companies in the sample period have violations and are investigated and punished by relevant regulatory authorities.

**Table 2:** Descriptive statistics.

Variables	Sample size	Mean	Standard deviation	Min	Median	Max
*Dona*	25,573	0.372	0.843	0	0.058	5.417
*Fraud*	25,573	0.088	/	0	0	1
*Size*	25,573	22.09	1.279	19.82	21.90	26.06
*Lev*	25,573	0.417	0.208	0.049	0.408	0.873
*ROA*	25,573	0.042	0.052	−0.175	0.039	0.188
*Cash*	25,573	0.988	1.752	0.025	0.405	11.60
*Dual*	25,573	0.277	/	0	0	1
*Bsize*	25,573	8.610	1.691	5	9	15
*Indep*	25,573	0.374	0.053	0.333	0.333	0.571
*Big4*	25,573	0.056	/	0	0	1

Table 3 presents the results of the single variable inspection. The average level of charitable donations of violating enterprises (*Fraud* = 1) is 0.376, which is significantly higher than that of non-violating enterprises (*Fraud* = 0). The results above show that enterprises that violate regulations will make higher charitable donations, thereby preliminarily validating Hypothesis 1.

**Table 3:** Single variable inspection.

Variable	*Fraud* = 0		*Fraud* = 1		Mean test
	*N*	Mean	*N*	Mean	
*Dona*	23,315	0.372	2,258	0.376	0.004***

*** indicates significance at the 1% level. *T*-test is used here.

### 5.2. Empirical result analysis

Table 4 reports the empirical test results of the impact of corporate violations on the level of charitable donations. The results show that the F-statistic of Eq. 1 is 19.6555, reaching the 1% significance level, thus indicating that the model design is reasonable, and the overall explanatory power is good. The coefficient of corporate violation (*Fraud*) is 0.040, which is significant at the 1% level. This indicates that there is a significant positive correlation between corporate violations and the level of corporate charitable donations—the level of charitable donations will increase significantly after corporate violations. Thus, Hypothesis 1 is verified. The regression results suggest that companies are largely motivated to use charitable donations to improve relationships with stakeholders in the wake of a breach, as well as to enhance their corporate image, reputation and ethical capital for insurance effect. In other words, charitable donations may cease to be purely altruistic and become a self-interested tool for some unethical companies to divert the attention of the capital market and conceal their violations.

**Table 4:** Corporate violation and charitable donation: benchmark regression results.

Variables	*Dona*
*Fraud*	0.055*** (3.00)
*Size*	0.012** (2.08)
*Lev*	−0.424*** (−11.11)
*ROA*	1.553*** (14.05)
*Cash*	0.019*** (5.31)
*Dual*	0.053*** (4.49)
*Bsize*	0.014*** (3.78)
*Indep*	0.333*** (2.97)
*Big4*	−0.121*** (−5.07)
*Constant*	0.231* (1.83)
*Ind*	Control
*Year*	Control
*N*	25,573
*R-squared*	0.075
*F-Statistic*	57.689***

***, **, and * indicate significance at the 1, 5, and 10% levels, respectively. The values in parentheses are *t*-statistics.

In terms of the regression results of the control variables, the coefficients of *Size*, *ROA* and *Cash* are all significantly positive. This indicates that the larger the firm size, the stronger the profitability, and the more abundant the cash flow, the more enthusiastic the enterprise is in philanthropic donations, which is consistent with the results of Brammer and Millington (2005) and Wang and Qian (2011). Moreover, *Lev* and *Big4* both have a significant negative correlation with corporate charitable donations, indicating that the higher the asset-liability ratio and audit quality, the lower the level of corporate charitable donations. This may be because when companies are facing greater repayment pressure and stronger external supervision, the benefits of corporate charitable donations are insufficient to offset their high cost. Hence, enterprises will reduce charitable donations. Additionally, the coefficients of *Dual*, *Bsize*, and *Indep* are all significantly positive. This may be because charitable donations help increase the value of the enterprise and profit for the business owner. Therefore, when the chairman is also the chief executive officer, the enterprise is more motivated to make charitable donations. Similarly, the larger the size of the board and the more independent directors, the more conducive it is for enterprises to absorb directors’ opinions on actively participating in charitable donations.

### 5.3. Robustness test

#### 5.3.1. Instrumental variable two-stage least squares method

There may be a two-way causal relationship between corporate violations and corporate charitable donations—enterprises with high donations are more likely to commit violations. Therefore, we used the average violation tendency of enterprises in the same industry and the same year (*Ind_Fraud*) and the average violation tendency of enterprises in the same province and the same year (*Pro_Fraud*) as the instrumental variables of enterprise violations. We re-estimated Eq. 1 using the instrumental variable two-stage least square method (IV 2SLS). The results are shown in Table 5, wherein Column (1) is the first-stage regression result, and Column (2) is the regression result of the second stage.

**Table 5:** Corporate violation and charitable donation: robustness test regression results.

Variables	IV 2SLS		PSM	Take firm-level effects into account	Change the measure of charitable donation
	(1)	(2)	(3)	(4)	(5)
	*Fraud*	*Dona*	*Dona*	*Dona2*	*Dona2*
*Ind_Fraud*	0.907*** (26.61)				
*Pro_Fraud*	0.881*** (22.54)				
*Fraud*		0.263*** (3.18)	0.044** (2.24)	0.033** (2.01)	0.079*** (3.65)
*Size*	−0.006*** (−3.33)	0.013** (2.34)	0.017* (1.82)	0.012* (1.90)	0.021*** (2.93)
*Lev*	0.091*** (7.14)	−0.445*** (−11.39)	−0.379*** (−6.42)	−0.265*** (−4.82)	−0.434*** (−8.69)
*ROA*	−0.534*** (−14.43)	1.679*** (13.87)	0.797*** (4.84)	0.882*** (7.18)	1.862*** (14.20)
*Cash*	0.001 (0.42)	0.019*** (5.24)	0.021*** (3.14)	−0.004 (−1.02)	0.037*** (5.35)
*Dual*	0.007* (1.79)	0.052*** (4.37)	0.046** (2.38)	0.010 (0.62)	0.070*** (4.60)
*Bsize*	−0.001 (−0.99)	0.014*** (3.84)	0.016** (2.48)	0.001 (0.15)	0.008* (1.65)
*Indep*	−0.025 (−0.67)	0.338*** (3.01)	0.584*** (3.08)	0.045* (1.72)	0.319** (2.24)
*Big4*	−0.017** (−2.15)	−0.116*** (−4.82)	−0.133*** (−2.78)	−0.049 (−0.98)	−0.142*** (−4.76)
*Constant*	0.115*** (2.71)	0.186** (2.07)	−1.015* (−1.65)	0.200 (0.60)	0.136* (1.83)
*Ind*	Control	Control	Control	Control	Control
*Year*	Control	Control	Control	Control	Control
*Firm*				Control	
*N*	25,569	25,569	9,264	25,573	18,566
*R-squared*			0.091	0.031	0.077
*F-Statistic*	651.24***	57.39***	9.71***	19.27***	45.36***
*Sargen, P-value*		0.137			

***, **, and * indicate significance at the 1, 5, and 10% levels, respectively. The values in parentheses are *t*-statistics.

Column (1) of Table 5 shows that the coefficients of the instrumental variables *Ind_Fraud* and *Pro_Fraud* are both significantly positive at the 1% level, and the F-statistic value is 651.24, which also reaches the 1% significance level, thus indicating that the model has good explanatory power. Column (2) shows that the value of *p* of the Sargan-statistic for testing the exogeneity of the instrumental variable is greater than 10%, indicating that the instrumental variables conform to the exogeneity assumption. More importantly, the coefficient of *Dona* is 0.263, which is significantly positive at the 1% level. This indicates that, after using instrumental variables to alleviate endogeneity, corporate violations are still significantly positively correlated with the level of corporate charitable donations, which is consistent with the research findings presented above.

#### 5.3.2. Propensity score matching method

Propensity score matching (PSM) (1: 4 nearest neighbor matching, where the matched variables in this paper are the control variables and all pass the balance test) is used to solve for endogeneity and reduce treatment selection bias in the fraud variable. Column (3) of Table 5 reports the results of the PSM test using the corporate violations (*Fraud*) as the grouping variable (1 for the treatment group and 0 for the control group), and it can be seen that the effect of corporate violations on the level of corporate charitable donations remains significantly positive under the PSM method estimation.

#### 5.3.3. Take firm-level effects into account

To ensure the robustness of the study’s findings, we have re-run the regression of the Model 1 taking into account firm-level fixed effects, the results of which are presented in Column (4) of Table 5. As can be seen, there is still a significant positive relationship between corporate violations and the level of corporate charitable donations when firm-level fixed effects are taken into account, validating the reliability of the findings.

#### 5.3.4. Change the measure of charitable donation

To reduce the impact of income volatility, we now take the ratio of corporate charitable donation amount to the three-year average operating income multiplied by 1,000 as the alternative variable of charitable donation, which is expressed by *Dona2*. It should be noted that the sample size was reduced due to the difference in indicators. The regression results are shown in Column (5) of Table 5. Apparently, the coefficient of *Dona2* is 0.079, which is significantly positive at the 1% level. This indicates that after changing the measurement method of charitable donation, there is still a significant positive correlation between corporate violations and the level of the corporate charitable donations, which verifies the robustness of our research conclusion.

## 6. Further research: heterogeneity analysis

The aforementioned research shows that enterprises will significantly improve their levels of charitable donations after violations to use the instrumental utility of charitable donations to divert the focus of the public and cover up their violations. In further research, through grouping regressions, we thoroughly explored whether internal and external factors, such as ownership type, degree of analyst attention and information transparency, affect the relationship between corporate irregularities and corporate charitable donations.

### 6.1. Ownership type, corporate violations, and corporate charitable donations

There are significant differences between state-and non-state-owned enterprises in terms of business environment and business objectives, as well as pressures of violations, the probability of being inspected and costs of violations. Thus, it can be inferred that the violations of enterprises of different ownership types may also have different impacts on corporate charitable donations. On the one hand, contrary to the natural advantages of state-owned enterprises in obtaining financing and government resources, non-state-owned enterprises usually face a poor operating environment and greater competitive pressure. Thus, they have a relatively strong incentive to commit a violation and the cost of doing so is high. Once their violations are discovered by the outside world, their production and operation will be severely affected. Moreover, it is generally difficult for non-state-owned enterprises to divert public attention and eliminate the negative impact of corporate violations through government channels, such as strengthening political ties. On the other hand, compared with state-owned enterprises, corporate violations will cause more direct losses to the management of non-state-owned enterprises, such as salary reductions and demotions. Based on their interests, the management of non-state-owned enterprises will be more inclined to use charitable donations to divert public attention and cover up the company’s irregularities. Therefore, we argue that non-state-owned enterprises have stronger incentives to use charitable donations to cover up their violations.

To examine the impact of ownership type on corporate violations and corporate charitable donations, we divided the sample into state-owned enterprises and non-state-owned enterprises for further examination. The results are shown in Columns (1) and (2) of Table 6. The results show that in non-state-owned enterprises, corporate violations can significantly improve the level of corporate charitable donations. However, there is no significant correlation between corporate violations and corporate charitable donations in state-owned enterprises. This may be because, as affiliated institutions under government leadership, state-owned enterprises generally have high social status and corporate reputation, relatively weak motives for violations and low violation costs. Moreover, state-owned enterprises can divert market attention through other means, such as political connections, without having to choose high-cost charitable donations to deal with the crisis caused by corporate non-compliance.

**Table 6:** Corporate violation and charitable donation: grouping regressions.

Variables	State-owned enterprises	Non-state-owned enterprises	Group with high analyst attention	Group with low analyst attention	Group with high information transparency	Group with low information transparency
	(1)	(2)	(3)	(4)	(5)	(6)
	Dona	Dona	Dona	Dona	Dona	Dona
*Fraud*	0.031 (1.29)	0.045* (1.83)	0.079*** (2.77)	0.014 (0.66)	0.114*** (3.72)	0.065** (2.33)
*Controls*	Control	Control	Control	Control	Control	Control
*Ind*	Control	Control	Control	Control	Control	Control
*Year*	Control	Control	Control	Control	Control	Control
*N*	9,197	16,378	12,871	13,118	7,926	7,926
*R-squared*	0.088	0.073	0.078	0.085	0.080	0.070
*F-statistic*	26.038	35.511	29.953	34.803	20.719	18.001

***, **, and * indicate significance at the 1, 5, and 10% levels, respectively. The values in parentheses are *t*-statistics.

### 6.2. Analyst attention, corporate violations, and corporate charitable donations

As professionals, analysts have a strong ability to collect information and integrate and transmit relevant information to investors promptly and effectively (Blankespoor and Dehaan, 2018). Analysts are usually prone to overreacting to good news, thus resulting in optimistic forecast bias (Easterwood and Nutt, 1999). This enables enterprises to take advantage of analysts’ information transmission roles to release good news actively. Therefore, we believe that the higher the analyst’s attention, the more motivated the violated enterprises are to make charitable donations to convey the positive image of the enterprise’s enthusiasm for public welfare to the outside world through analysts, thereby suppressing the negative information of the enterprise.

To test whether there are differences in the impact of corporate violations on corporate charitable donations under different levels of analyst attention, we referred to the common practice of the existing literature (Yu, 2008; Irani and Oesch, 2016). We used the number of analysts tracking a listed company as an indicator to measure the attention of enterprises by analysts. Grouping regressions were conducted according to whether analysts pay more attention to the company than the median of the same industry and year. The regression results are shown in Columns (3) and (4) of Table 6. The results show that in groups with a high degree of analyst attention, corporate violations are significantly positively correlated with the level of charitable donations at the 1% level. In groups with a low degree of analyst attention, there is no significant correlation between corporate violations and the level of corporate charitable donations. This may be because analysts have the function of transmitting information. When an enterprise is highly concerned by analysts, stakeholders perceive the charitable donation behavior of the enterprise promptly and fully, attract extensive attention from the capital market, and bring high reputation benefits to this enterprise to cover up the enterprise’s violations effectively. Therefore, to maximize benefits, violated companies highly concerned with analysts will make more charitable donations.

### 6.3. Information transparency, corporate violations, and corporate charitable donations

On the one hand, when enterprises have high information transparency—a low degree of information asymmetry—the market is more likely to detect the violations of enterprises and produce serious negative reactions. Therefore, the violation costs and investigation risks of enterprises are relatively high at this time. On the other hand, companies with poor information transparency can compensate for their violations through low-cost means, such as earnings management, and they will not be easily detected by the outside world (Hutton et al., 2009). Therefore, for cost-effectiveness, enterprises with high information transparency levels are more inclined to divert the public’s attention to their violations through charitable donations.

Referring to Hutton et al. (2009), we used the three-year cumulative modified Jones coefficient as the variable to measure enterprise information transparency, and the larger the value, the lower the enterprise information transparency. Grouping regressions were conducted according to whether the cumulative revised Jones coefficient of the enterprise in 3 years is greater than the median of the same industry and year. It should be noted that the sample size is reduced as the variable is calculated using multi-year data. The results are presented in Table 6. The regression results show that among the groups with high information transparency, the coefficient of enterprise violation is 0.114, which is significant at the 1% level. Among groups with low information transparency, the coefficient of enterprise violation is 0.065, which is significant at the level of 5%. It can be found that in the groups with high information transparency, the positive relationship between corporate violations and charitable donations is more significant. This shows that enterprises, as rational organizations, will consider the principle of cost-effectiveness when using charitable donations as a tool to conceal negative information. Violated enterprises will adopt the means of charitable donation only when information transparency is high, and it is difficult to divert external attention through other ways.

## 7. Conclusion and suggestions

The motivation behind corporate charitable donations is a trending topic in academia. In recent years, there has been a boom in corporate charitable giving in China’s capital markets, which is accompanied by frequent corporate violations. In order to explore the motives of corporate charitable donations, specifically, to reveal whether corporate charitable donations in China’s capital market are made for the undesirable purpose of concealing information about violations, the present study empirically investigated the influence of corporate violations on corporate charitable donation behavior, using a sample of 3,715 non-financial companies in Chinese A-shares from 2011 to 2020. Further, we examined the heterogeneous impact of ownership types, analyst attention, and information transparency among them to reveal the motivation behind corporate charitable donations against the background of corporate violations. Herein, the study’s outcome confirmed that enterprises make more charitable donations after violation, and this effect is more significant in non-state-owned enterprises, listed companies with high analyst attention and high information transparency. The research results show that corporate charitable donations may act as a distraction from external focus and a cover-up for corporate violations, thus violated companies have a strong incentive to make instrumental charitable donations. Specifically, in order to mitigate the severe negative impact when the corporate violation is revealed, companies will significantly increase their charitable giving after the violation to enhance the trust of their stakeholders and create reputational capital, thus making it to be insurance against rainy days. Furthermore, violating enterprises will fully consider the cost–benefit principle when making charitable donations based on instrumental motives, which enriches the relevant research on the instrumental motivation behind charitable donations and provides new ideas for follow-up research.

Unlike previous studies in developed countries, this study is based on panel data from a developing country, namely China. Moreover, this paper presents an innovative study of the hidden motives behind corporate charitable giving from the perspective of corporate violation. The results of this study are expected to deepen the understanding of corporate charitable donations in China’s capital market and provide new insights to optimize the ecology of China’s capital market and help investors in their decision-making. The research conclusions may have certain enlightening significance for government departments and investors.

First, charitable donations may become a tool for some enterprises to conceal their violations, and from this perspective, corporate charitable donations are somewhat hypocritical. Therefore, while encouraging companies to actively fulfill their social responsibilities, the government should establish and improve the information disclosure system for corporate charitable donations. It should strengthen the regulation and guidance of corporate charitable donations to help enterprises, especially non-state-owned enterprises, establish the correct concept of charitable donation. Additionally, cost-effectiveness is the key principle that enterprises consider when making charitable donations based on instrumental motivation. When benefits are insufficient to offset costs, the enterprise will consciously reduce or even give up charitable donations for instrumental purposes. Therefore, the government can reduce the effect of corporate charitable giving in diverting the public’s attention and concealing non-compliant information by disseminating relevant knowledge at the social level, alerting and calling on investors, consumers, the public and other stakeholders to pay attention to the hidden motives behind corporate charitable giving, thereby combating the tendency of corporate instrumental giving and achieving the long-term development of the capital market.

Second, investors should have a more comprehensive, rational, and objective understanding of corporate charitable donations. With the increasingly fierce competition in China’s capital market in recent years, charitable donations are no longer for purely altruistic or simple strategic purposes. They may also be used by enterprises as tools to cover up irregular acts and reduce their losses. Behind the generosity of companies, there may be hidden violations that are not yet known. In non-state-owned enterprises, listed companies with high analyst attention and high information transparency, the instrumental motivation behind the charitable donation of violating enterprises is stronger. Investors should therefore improve their judgment and rationality through extensive study, and view corporate charitable giving from a more comprehensive, rational and objective perspective. In the face of listed companies with frequent large donations, especially non-state-owned enterprises that have received high attention from analysts, investors should be more cautious and conduct a detailed as well as comprehensive investigation into the donation background and development status, focusing on the motives of the company’s charitable donations so as not to be confused by the appearance of charity and make wrong investment decisions.

## Data availability statement

The original contributions presented in the study are included in the article/supplementary material, further inquiries can be directed to the corresponding author.

## Author contributions

QZ, FY, and CW contributed to conception and design of the study. DL organized the database. HW performed the statistical analysis. QZ wrote the first draft of the manuscript. QZ, FY, HW, and CW wrote sections of the manuscript. All authors contributed to manuscript revision, read, and approved the submitted version.

## Funding

This work was supported by the Humanities and Social Sciences Fund of the Ministry of Education (Grant No. 21YJA790076).

## Conflict of interest

The authors declare that the research was conducted in the absence of any commercial or financial relationships that could be construed as a potential conflict of interest.

## Publisher’s note

All claims expressed in this article are solely those of the authors and do not necessarily represent those of their affiliated organizations, or those of the publisher, the editors and the reviewers. Any product that may be evaluated in this article, or claim that may be made by its manufacturer, is not guaranteed or endorsed by the publisher.
